# Sorafenib-Associated Heart Failure Complicated by Cardiogenic Shock after Treatment of Advanced Stage Hepatocellular Carcinoma: A Clinical Case Discussion

**DOI:** 10.1155/2017/7065759

**Published:** 2017-04-27

**Authors:** Candace Wu, Kamal Shemisa

**Affiliations:** ^1^University of Texas Southwestern Medical Center, Dallas, TX, USA; ^2^Division of Cardiology, Department of Internal Medicine, University of Texas Southwestern Medical Center, Dallas, TX, USA

## Abstract

*Background.* Sorafenib, an oral tyrosine kinase inhibitor (TKI), targets multiple tyrosine kinase receptors (TKRs) involved in angiogenesis and tumor growth. Studies suggest that inhibition of TKR impacts cardiomyocyte survival. Inhibition of VEGF signaling interrupts angiogenesis and is associated with the development of hypertension and compensatory hypertrophy. Compensated hypertrophy ultimately leads to heart failure.* Case Description.* A 76-year-old man with a past medical history of systolic heart failure due to ischemic cardiomyopathy and stage IIIC hepatocellular carcinoma (HCC) presented with symptoms of decompensated heart failure. Four months prior to admission, he was started on sorafenib.* Results*. Our patient was treated with intravenous furosemide and guideline directed therapy. Clinical status was complicated by the development of low cardiac output and shock requiring inotropic support. Careful titration of heart failure medication led to hemodynamic improvement and discontinuation of dobutamine.* Conclusion*. Greater awareness of sorafenib cardiotoxicity is essential. As TKI usage grows for treatment of cancers, heart failure-related complications will increase. In our patient, routine heart failure management and cessation of sorafenib led to clinical improvement. Future studies on the treatment of sorafenib cardiotoxicity should be explored further in this unique patient population.

## 1. Introduction

Sorafenib is an oral multikinase inhibitor that targets pathways involved in angiogenesis and tumor growth by inhibiting tyrosine kinase receptors and is recommended as a first-line drug for the treatment of advanced hepatocellular carcinoma due to limited alternatives. The effect of sorafenib on the human body is not limited to hepatocellular cancer cells and has been linked to cardiovascular toxicity leading to the pathogenesis of clinical heart failure and resistant hypertension [1, 2].

This report presents the clinical case of a patient with ischemic cardiomyopathy and advanced HCC who was started on sorafenib and subsequently developed heart failure complicated by cardiogenic shock. We will review the clinical case, the biological basis for TKI cardiac toxicity, and the therapeutic challenges and considerations for the treatment of sorafenib-associated hypertension and heart failure.

## 2. Clinical Case

**Figure 1:**
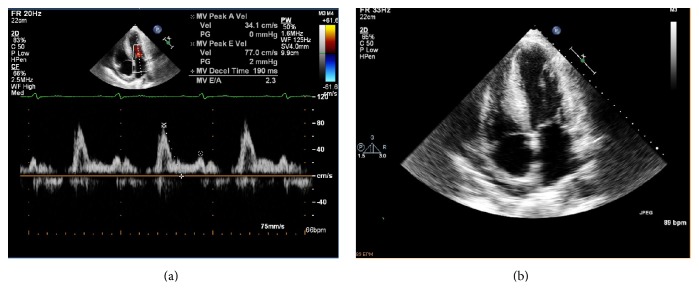
(a) At baseline, the transthoracic echocardiogram demonstrates moderately depressed LV function (LVEF-40%) with significant concentric and septal LV hypertrophy (LV mass 367 grams, IVSd-2.1 cm, PWT-1.5 cm, RWT-0.67). (b) There is further evidence of elevated filling pressures. There is left atrial enlargement (LA dimension-4.6 cm), mildly elevated estimated right ventricular systolic pressure (RVSP~36 mmHg), and restrictive filling on mitral inflow Doppler (E/A-2.3, deceleration time 190 milliseconds).

A 76-year-old man presented with worsening dyspnea at rest and on exertion, orthopnea, and lower extremity edema. The past medical history was significant for single-vessel coronary artery disease (s/p percutaneous coronary intervention), systolic heart failure with reduced ejection fraction (LVEF 37%) due to ischemic cardiomyopathy, severe ventricular hypertrophy, chronic atrial fibrillation, hypertension, hyperlipidemia, diabetes mellitus type two, chronic obstructive pulmonary disease, and Stage IIIC hepatocellular carcinoma. Family history was positive for hypertension and diabetes mellitus type two. He was previously treated with guideline directed therapy for systolic heart failure (Table 1). The transthoracic echocardiogram was significant for moderately depressed left ventricular (LV) function, left atrial dilation, normal ventricular chamber dimensions, increased LV mass, and global hypokinesis (Figure 1).

Eight months prior to admission, a cardiac MRI demonstrated findings suggestive of mild inferior wall myocardial ischemia on gadolinium imaging and moderately depressed systolic dysfunction. He was treated medically for ischemic heart failure without further risk stratification. Four months prior to admission, the patient was incidentally found to have a palpable right upper quadrant mass, which, by ultrasound, revealed a large heterogeneous echogenic density (measuring 13 cm). He underwent transhepatic biopsy and pathologic results were significant for poorly differentiated pleomorphic hepatocellular carcinoma. CT of the abdomen and pelvis was significant for tumor extension into the inferior vena cava and subdiaphragmatic space. A markedly elevated alpha-fetoprotein (AFP) level (>10,000 ng/mL) was measured, suggestive of a high tumor burden.

His WHO cancer staging was Stage IIIC based on clinical as well as imaging findings. The 1-year survival of Stage IIIC HCC is 6–8 months for patients and the recommended treatment is a tyrosine kinase inhibitor (TKI) called sorafenib, which extends median survival length to 10.7 months [3].

**Figure 2:**
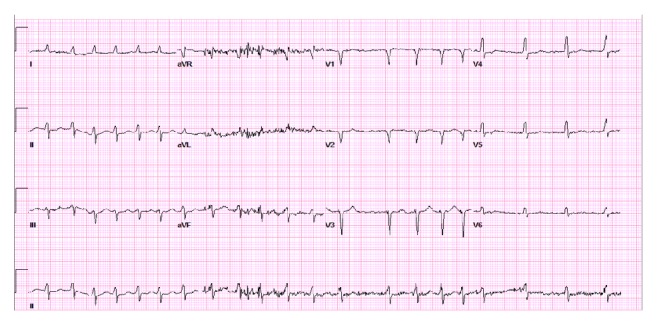
Atrial fibrillation with rapid ventricular response (ventricular rate 127 bpm) with evidence of prior septal infarct and nonspecific ST-T wave abnormalities in the precordium. There are low QRS voltages noted across leads likely due to chronic obstructive pulmonary disease.

The patient presented to the emergency department hypertensive (169/79 mmHg) and tachycardic with a ventricular rate of 127 beats per minute (Figure 2). The physical exam was consistent with volume overload, and admission labs were notable for elevated BNP, abnormal BUN/creatinine, and elevated liver function tests (Table 2). Chest X-ray demonstrated cardiomegaly with pulmonary venous congestion and a moderate-sized right-sided pleural effusion.

**Figure 3:**
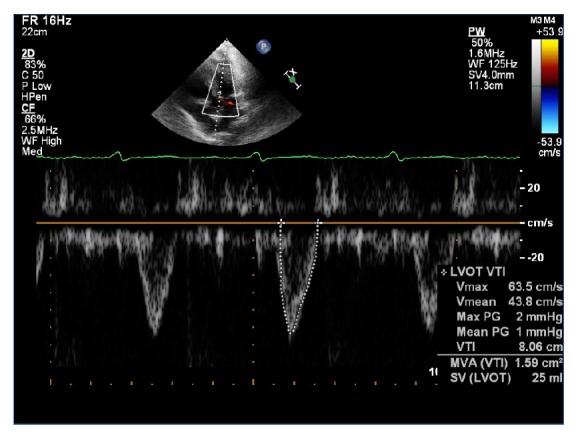
At the time of clinical deterioration, pulse Doppler of the LVOT and continuity equation calculated SV/CO (LVOT dimension-2.0 cm, VTI-8.1 cm) suggested low stroke volume (25 ml) and cardiac output (2.22 L/min).

**Figure 4:**
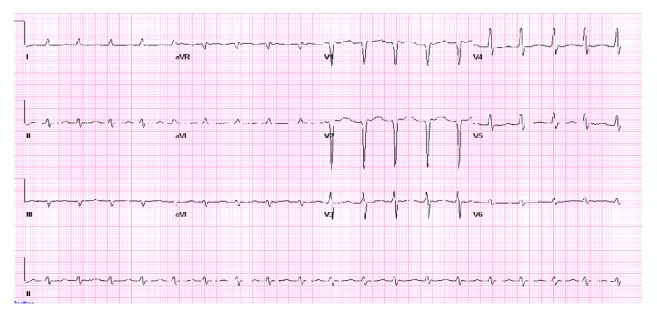
Atrial fibrillation with fairly regular R-R intervals (ventricular rate 113 bpm) and slower rate while on beta-blocker. The QRS morphology and voltages remain unchanged from prior tracing.

The clinical exam was consistent with decompensated heart failure. The patient was treated with intravenous furosemide, carvedilol, and lisinopril. After sufficient diuresis (10 liters of fluid), clinical symptoms improved minimally (Table 3). However, within the subsequent 18 hours, the patient developed persistent hypotension (MAP 60 mmHg), worsening renal function, and cool extremities indicative of impending cardiogenic shock. He continued to have elevation of JVP on physical exam, which suggested restrictive filling of the right ventricle (RV) as well as RV failure. A pulse wave Doppler across the LVOT suggested diminished stroke volume after calculation of the LV velocity time integral (Figure 3). A right heart catheterization was subsequently performed which showed mildly elevated right- and left-sided ventricular filling pressure, pulmonary hypertension, and low cardiac output (Table 3). He was then treated with continuous infusion of dobutamine and furosemide. An EKG at the time demonstrated improvement of the ventricular rate without evidence of ST-T wave changes (Figure 4).

Based on the antecedent history of recent initiation of sorafenib followed by deterioration in cardiac function, sorafenib-induced decompensated heart failure was diagnosed. This case report serves as an example of the therapeutic challenges of heart failure management in patients treated for hepatocellular carcinoma with sorafenib. The cardiac toxicity of sorafenib is known and while many patients who have preexisting heart failure are not initiated on tyrosine kinase inhibitors for this reason, limited therapies for cancers such as HCC may compel care providers to do so. Cautious clinical decision-making, heart failure follow-up, and recognition of higher risk groups in this unique subset of patients are needed. It is our goal to discuss the mechanism of sorafenib-associated cardiac toxicity and other tyrosine kinase inhibitors. We also review clinical strategies for treatment of heart failure complications.

## 3. Discussion

Sorafenib is an oral tyrosine kinase inhibitor that targets multiple tyrosine kinase receptors involved in angiogenesis and tumor growth and is recommended as a first-line drug for the treatment of advanced HCC [1]. In cancer cells, tyrosine kinase activity is increased due to mutations in the genes associated with the fundamental regulatory components of the enzyme. Mutations in cancer cells can also lead to overexpression of the tyrosine kinase genes. Tyrosine kinase inhibition is mediated through binding of a ligand or antibody (inhibitor), thus preventing the receptor from achieving its activated form. Autophosphorylation of the tyrosine kinase subsequently activates a series of protein kinase cascades in one of two dominant pathways involved in cell proliferation, hypertrophy, function, and survival. These are the phosphatidylinositol 3-kinase (PI3K) pathway via AKT or the mitogen-activated protein kinase (MAPK) cascade via RAS and ERK (extracellular signal regulated kinase) [4, 5].

*The Signaling Pathway of Tyrosine Kinases, Sites of Inhibition, and Cardiotoxicity Secondary to Trastuzumab following Treatment of HER2+ Breast Cancer*. To further understand the reasons for sorafenib-associated cardiotoxicity, it is relevant to discuss the lessons learned from earlier generation TKIs, the exact cellular pathways affected, and the mechanisms behind the pathogenesis of clinical heart failure. Patients with ERBB2 receptor, also known as HER2 (human epidermal growth factor receptor), breast cancer that were receiving anthracycline-based chemotherapy were 3.5 times more likely (8% versus 27%, *p* < 0.001) to develop asymptomatic cardiac dysfunction on echocardiography or clinical heart failure [6]. Clinically significant predictors for cardiac toxicity among several cohorts receiving trastuzumab were history of cardiac disease at baseline, treatment with anthracyclines, and age > 70 years [7, 8].

The true incidence of heart failure secondary to trastuzumab alone was likely unknown due to treatment confounding with anthracyclines and taxanes. Assessing the true clinical risk of cardiomyopathy among patients receiving trastuzumab has therefore led to clinical trials with prespecified cardiac end points which estimate the incidence to be less frequent when administered alone (4–7%) [9, 10]. Clinical follow-up data from these trials further demonstrated that trastuzumab cardiac toxicity is reversible shortly after discontinuation and may be responsive to medical therapy [11]. This would suggest that the severity of cardiac toxicity secondary to TKIs is most pronounced when the cardiac myocyte is simultaneously weakened by another process that creates an energy deficit, and, subsequently, the required metabolic signaling needed to maintain survival is hindered through the disruption of these critical pathways. As will be described, the degree of cardiac toxicity caused by TKIs depends on the inhibitory effect on the dominant signaling pathway of the cardiac myocyte, the selectivity of tyrosine kinase inhibition, the concomitant treatment with older cardiotoxic chemotherapeutic agents, anthracyclines or taxanes, and the duration of treatment [10, 12]. 

*Tyrosine Kinase Inhibition Promotes an Energy Deficit and Interferes with Cardiac Myocyte Survival Pathways*. TKIs are considered very effective in the treatment of the respective cancers but, as was described, can have deleterious effects on the cardiovascular system. Both clinical experiences followed by basic investigations regarding deficient cellular signaling due to trastuzumab and bevacizumab have furthered our understanding of the importance of tyrosine kinase signaling in the pathogenesis of heart failure and hypertension in patients receiving these treatments for cancer. As newer TKIs are being developed, there is greater scrutiny for reporting adverse cardiovascular events during clinical trials. In the search for better and more effective anticancer drugs, multikinase inhibitors were developed to achieve greater inhibition of TKR and downstream signaling. The ultimate effect of less selectivity is the shutting down of signaling, ATP utilization, and the cell machinery for cell division. Sorafenib, a multikinase inhibitor, was designed for the inhibition of cell proliferation.

**Figure 5:**
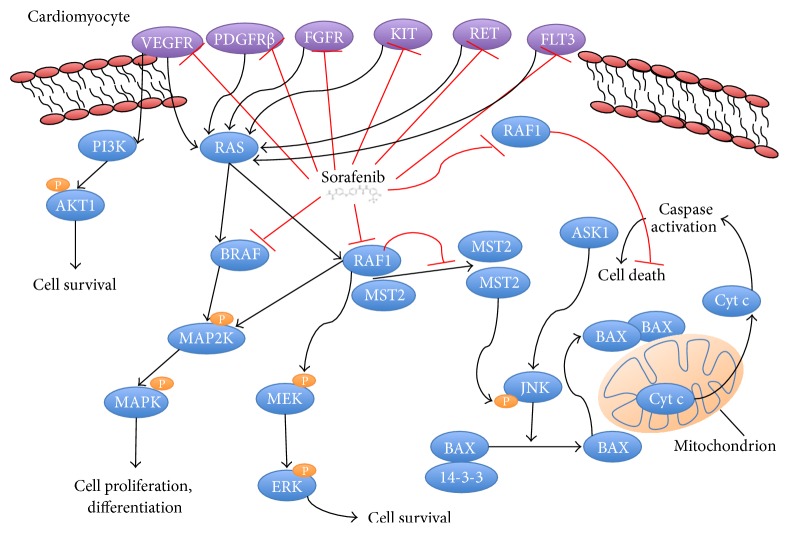
Sorafenib inhibits multiple tyrosine kinase and second messenger pathways. In the cardiac myocyte, inhibition of prosurvival pathways RAS/MAPK via BRAF and P13K/AKT1 via VEGFR leads to diminished survival. Sorafenib further inhibits RAF1, which leads to loss of tonic inhibition of cell death pathways and death through activation of caspases.

Studies that have elucidated the cellular mechanisms behind cardiotoxicity due to sorafenib suggest that inhibition of RAF-1 and B-RAF kinase impact cardiomyocyte survival (Figure 5) [12]. RAF-1 signaling phosphorylates a series of kinases in the MAPK group, MEK1 and ERK1, which potentiates cardiac myocyte hypertrophic growth and survival [13, 14].

The RAF-1 kinase also inhibits cell death pathways mediated by apoptosis signal-regulating kinase 1 (ASK1) as well as mammalian sterile 20 kinase 2 (MST2). Both ASK1 and MST2 are proapoptotic kinases and have roles in oxidant stress-induced injury. Deletions of RAF-1 in the heart result in ventricular dilation, reduced contractility, and increased cell death [12, 15]. When mice with deleted RAF-1 are subjected to pressure overload, cardiomyocyte death was increased, indicating that RAF-1 kinase activity may be cardioprotective [16].

Nonselective TKIs such as sorafenib that target multiple kinases have great potential for intracellular toxicity. Myocardial cells require greater energy demands than other cells and therefore are particularly sensitive to deficiencies in energy production. Some case reports have indicated the potential for reversibility of sorafenib-induced cardiomyopathy after discontinuation though it is unclear to what extent or after what duration of treatment that reversibility is still feasible [17].

*Mechanism of Cardiotoxicity after Treatment with Sorafenib May Be In Part due to VEGF Inhibition*. TKRs were initially noted to enhance gene expression and protein synthesis involved in cardiac hypertrophy [18]. Deleterious effects on the cardiovascular system were observed with inhibition of VEGF (vascular endothelial growth factor) and PDGF (platelet derived growth factor) receptors. Inhibition of VEGF signaling with bevacizumab was shown to increase vascular tone and disrupt microvascular blood flow [19, 20]. Clinical consequences of disrupting VEGF signaling include a higher risk for worsening hypertension and a twofold greater risk for heart failure among patients with cancers of solid tumor origin [21, 22]. Among patients undergoing treatment of renal cell carcinoma (RCC) with sorafenib, 68% had some form of cardiovascular toxicity, with most cases (59%) having worsened hypertension. Newly diagnosed heart failure, defined as an elevation in NT-proBNP and a decline in LVEF, occurred in 40% of patients on sunitinib and in 4% of patients on sorafenib [2]. In a meta-analysis of 21 randomized controlled trials with patients undergoing treatment of HCC, there was a 2.7-fold greater heart failure risk in those that received TKIs compared to those who did not (2.39% versus 0.75%, *p* < 0.001) [23]. 

*Therapeutic Challenges and Considerations in the Treatment of Hypertension*. Hepatocellular carcinoma is one of the most common cancers in the world and typically presents in advanced stages. Standard treatments during early stages include partial hepatectomy, local ablation, and liver transplantation. Sorafenib is recommended as a first-line drug for the treatment of advanced HCC [1, 2].

**Figure 6:**
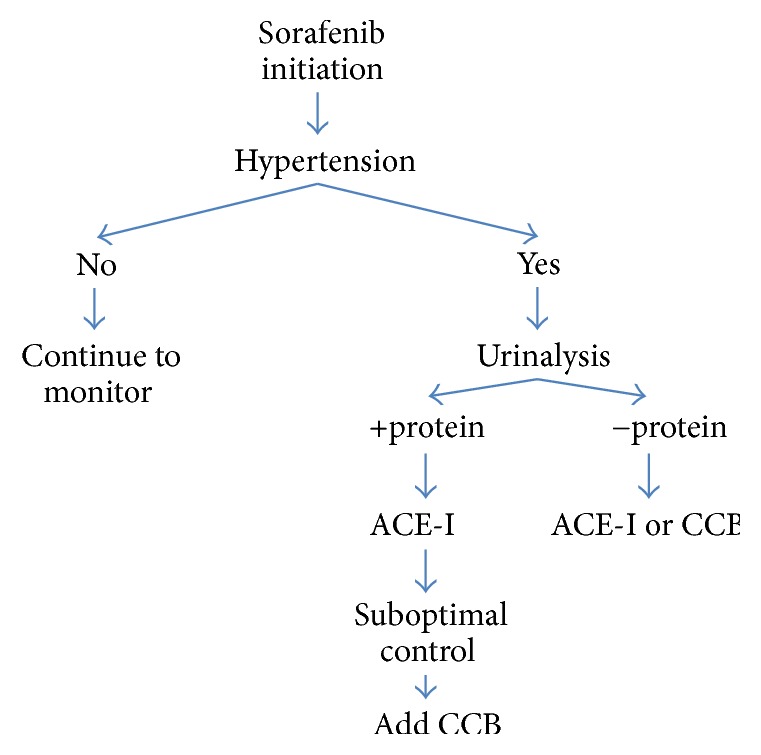
Algorithm for treatment of sorafenib-induced hypertension.

In the case of sorafenib-induced hypertension, blood pressure is treated with conventional therapy and appears not to impact or worsen progression-free survival among patients treated for metastatic RCC [24]. Early intervention of hypertension is essential to reduce subsequent risks of developing cardiomyopathy and heart failure. The Angiogenesis Task Force of the National Cancer Institute Investigational Drug Steering Committee recommends regular blood pressure monitoring before treatment as well as during treatment, weekly during the first cycle and then every 2-3 weeks, with a goal BP of less than 140/90 mmHg for most patients and a goal BP of less than 130/80 for those with diabetes or chronic kidney disease [25]. Sorafenib is also associated with the development of proteinuria, and it is recommended that urine protein be tested before and after treatment initiation. When patients develop proteinuria, clinicians should strive to control blood pressure with either angiotensin-converting enzyme inhibitors (ACE-I) or dihydropyridine calcium channel blockers (CCB), which have been recommended as first- and second-line agents (Figure 6) [26]. Treatment with ACE-I is contraindicated if patients develop hyperkalemia, severe cough, or angioedema or have advanced chronic kidney disease. Transitioning to angiotensin receptor blocker is possible in those patients intolerant to ACE-I though clinical data is limited. Discontinuation of sorafenib can be considered in patients with resistant hypertension.

*Therapeutic Challenges and Considerations in the Treatment of Systolic Heart Failure*. Although it is typically recommended that chemotherapy be discontinued when significant heart failure develops, case reports have shown that sorafenib can be used to treat advanced HCC for as long as 12 months and may be reduced in those that develop heart failure [1]. It is difficult to know which heart failure medications would benefit patients exposed to TKIs. Very few clinical studies have investigated the treatment of heart failure due to TKIs in a prospective fashion. Newer generation TKIs are less selective than their predecessors, and while the cancer cell growth is strongly halted, the potential for cardiac toxicity is higher. At this time there are no medications that specifically target and reverse TKI-related cardiotoxicity. The ACC/AHA guidelines recommend that patients receiving chemotherapeutic agents should be monitored carefully. A baseline echocardiogram prior to treatment with TKIs in patients with cardiac risk factors and close follow-up in monitoring for heart failure symptoms is appropriate [27, 28].

In patients that develop clinical heart failure or systolic dysfunction on echocardiography, treatment with a combination of guideline directed ACE-I, and beta-blockers have been shown to reduce heart failure-associated mortality and improve cardiovascular morbidity [27]. The most well studied agents in the treatment of anthracycline-induced cardiomyopathy are carvedilol and enalapril though the mechanism, severity, and toxicity of anthracyclines differ from the cardiotoxicity of TKIs [29, 30]. Aldosterone antagonists have also been shown to further reduce mortality in advanced heart failure in patients with systolic heart failure, LVEF < 35%, and NYHA III-IV. Treatment of salt and water retention in systolic heart failure is further treated with loop diuretics. Implantable defibrillators used as primary prevention for sudden cardiac death are recommended in those with greater than a 1-year life expectancy with persistently severe LV dysfunction for greater than three months despite optimal medical therapy. In patients that develop heart failure symptoms and successfully complete treatment with TKIs, follow-up echocardiography is appropriate to assess for reversal of LV dysfunction [26]. 

*Dobutamine Treatment May Be Considered in Those with Severe Heart Failure*. Baseline diminished contractile reserve with subsequent exposure to sorafenib led to the development of cardiogenic shock in our patient who was subsequently treated with dobutamine, an inotrope with weaker chronotropic effects, which successfully restored a normal hemodynamic profile. While it is well known that inotropic support is associated with adverse long-term cardiovascular outcomes, the clinical efficacy of inotropes after exposure to cardiac toxic medications is unknown. Dobutamine's pharmacologic function does not depend on tyrosine kinases but rather on G-protein coupled receptors. Whether sensitivity to beta-1 adrenergic activation by dobutamine is increased after tyrosine kinase inhibition is unknown. In response to isoproterenol, the presence of genistein was shown to potentiate beta-adrenergic sensitivity in the heart of animal models [31].

## 4. Conclusion 

In our patient with underlying ischemic cardiomyopathy and advanced hepatocellular carcinoma, the management of heart failure in the setting of coexisting malignancy poses interesting and new clinical challenges. Sorafenib is a tyrosine kinase inhibitor that targets multiple tyrosine kinase receptors involved in angiogenesis and tumor growth. Unfortunately, sorafenib-associated cardiotoxic effects such as hypertension, reduced LVEF, and heart failure are of great concern and contribute to the challenging nature of HCC management. It is essential that TKI-induced hypertension be treated aggressively with standard treatments and regular monitoring. Guidelines also recommend echocardiogram monitoring for patients started on chemotherapeutic agents, especially those with cardiac risk factors, prior to treatment initiation, once heart failure symptoms develop, and after treatment completion. There are currently no specific recommendations for managing TKI-induced heart failure, and patients are typically managed with standard heart failure treatments. Beta-adrenergic effects of dobutamine may actually be potentiated by tyrosine kinase inhibition.

We initially considered more common causes for heart failure exacerbation in our patient. Patients with restrictive heart failure often develop worsened heart failure symptoms due to sustained periods of atrial fibrillation with rapid ventricular response. Prior to hospitalization, however, the patient's ventricular rate was well controlled with beta blockade, and it is our belief that the interval decline in ventricular function led to increased left atrial pressure and presentation of atrial fibrillation with rapid ventricular response. Furthermore, there were no EKG abnormalities suggestive of myocardial infarction or evidence of severe valvular regurgitation on echocardiography. There was the possibility that overdiuresis due to an inability to accurately estimate the filling pressures on physical exam led to inadequate preload leading to low cardiac output shock. However, the patient's right heart catheterization reflected that the filling pressures remained elevated after diuresis and that the etiology of low cardiac output was mainly attributed to diminished contractility.

After successful titration of guideline directed treatment and discontinuation of dobutamine, a repeat echo demonstrated baseline LV function. A repeat trial of sorafenib at a lower dose was offered to the patient but was declined. Although sorafenib is the potential predisposing factor for the patient's decompensation, the causal relationship between this drug and heart failure is still unclear since rechallenge was not performed. The patient was discharged home with palliative care follow-up for coordination of hospice.

## Figures and Tables

**Table 1:** The pertinent medication list and dose.

Medication	Dose
Sorafenib	200 mg by mouth twice daily
Carvedilol	3.125 mg by mouth twice daily
Furosemide	80 mg by mouth twice daily
Lisinopril	10 mg by mouth daily
Aspirin	81 mg by mouth daily
Warfarin	2.5 mg by mouth daily
Nitroglycerin	0.4 mg sublingual as needed

**Table 2:** Admission physical exam and notable labs.

Physical exam	Vital signs	BP 169/79 mmHg, HR 129 bpm	
	Cardiovascular	Irregularly irregular rhythm, tachycardic, JVP 15 cm H_2_O with head of bed at 30 degrees	
	Lungs	Symmetric chest rise with bibasilar rales	
	Extremities	3+ lower extremity pitting edema to the mid-shins bilaterally	

		Patient result	Normal

Laboratory results	Sodium	139	135–145 mEq/L
	Potassium	4	3.6–5.0 mEq/L
	BUN	21	7–21 mg/dL
	Creatinine	1.48	<1 mg/dL
	Magnesium	1.6	1.4–1.8 mEq/L
	BNP	3298	<100 pg/mL
	Troponin I	0.05	<0.03
	AST	95	5–35 U/L
	ALT	60	7–56 U/L
	Alkaline phosphatase	234	38–126 U/L
	Total bilirubin	4.2	0.2–1.3 mg/dL
	Procalcitonin	**+**	Undetectable

**(a):** 

Physical exam	Vital signs MAP 59–62 mmHg, HR 92 bpm, RR 20/min	Right heart catheterization	Measurements
General	Wrapped in blankets including head. Appears fatigued.	RA mean pressure	8 mmHg
Head, neck, throat	Dry mucous membranes.	RV pressure	33/9 mmHg
Cardiovascular	Irregularly irregular. Parasternal holosystolic murmur. +RV gallop. JVP 20 cm H_2_O at 30 degrees.	PA pressure	32/23 mmHg, mean 27 mmHg
Lungs	Coarse breath sounds. No wheezes. Normal work of breathing.	PCWP	18 mmHg
Abdomen	Soft, normoactive bowel sounds. Moderate to severe hepatomegaly with hepatojugular reflux.	Pulmonary vascular resistance	3.8 WU
Extremities	2+ pitting edema to knees. No cyanosis or clubbing.	Cardiac output (L/min) (thermal dilution)	3.91 (L/min), Sv02-53%, Hgb 15.2
Skin	Slightly cool extremities.	Cardiac index	1.70 L/min/m^2^

**(b):** 

Physical exam	Vital signs MAP 72 mmHg, HR 85 bpm, RR 14/min	Right heart catheterization	Measurements
General	Brighter affect.	RA mean pressure	5 mmHg
Head, neck, throat	Moist mucous membranes.	RV pressure	26/5 mmHg
Cardiovascular	Irregularly irregular. Parasternal soft holosystolic murmur. JVP 8 cm H_2_O at 30 degrees.	PA pressure	26/15 mmHg, mean 19 mmHg
Lungs	Clear breath sounds. No wheezes. Normal work of breathing.	PCWP	12 mmHg
Abdomen	Soft, normoactive bowel sounds. Moderate hepatomegaly with firm liver edges, absent hepatojugular reflux.	Pulmonary vascular resistance	1.4 WU
Extremities	No edema, cyanosis, or clubbing.	Cardiac output (L/min) (thermal dilution)	5.3 (L/min), Sv02-75%, Hgb 14.2
Skin	Pink fingertips and warm extremities.	Cardiac index	2.4 L/min/m^2^

## Abbreviations

ACC: American College of Cardiology
ACE-I: Angiotensin-converting enzyme inhibitors
AFP: Alpha-fetoprotein
AHA: American Heart Association
ASK1: Apoptosis signal-regulating kinase 1
BP: Blood pressure
BPM: Beats per minute
CCB: Calcium channel blocker
EGFR: Epidermal growth factor receptor
ERK: Extracellular signal regulated kinase
HCC: Hepatocellular carcinoma
HER2: Human epidermal growth factor receptor
JVP: Jugular venous pressure
LV: Left ventricle
LVEF: Left ventricular ejection fraction
MAP: Mean arterial pressure
MAPK: Mitogen-activated protein kinase
MST2: Mammalian sterile 20 kinase 2
MRI: Magnetic resonance imaging
NT-proBNP: N-terminal prohormone of brain natriuretic peptide
NYHA: New York Heart Association
PA: Pulmonary artery
PCWP: Pulmonary capillary wedge pressure
RAF1: Rapidly accelerated fibrosarcoma
RCC: Renal cell carcinoma
RHC: Right heart catheterization
RV: Right ventricle
TACE: Transcatheter arterial chemoembolization
TKI: Tyrosine kinase inhibitor
TKR: Tyrosine kinase receptor
VEGF: Vascular endothelial growth factor
VEGFR: Vascular endothelial growth factor receptor
WHO: World Health Organization.
